# Family hardiness among primary caregivers of breast cancer patients in Hunan Province: a cross-sectional study exploring the relationship with attachment and caregiver preparedness

**DOI:** 10.3389/fpubh.2024.1367029

**Published:** 2024-11-01

**Authors:** Xin Sun, Lili Xu, Lijuan Sheng

**Affiliations:** ^1^School of Nursing, Changsha Medical University, Changsha, China; ^2^Department of Breast Surgery, The Second Xiangya Hospital, Central South University, Changsha, China; ^3^The Second Xiangya Hospital, Central South University, Changsha, China

**Keywords:** caregiver, breast cancer, family hardiness, experiences in close relationships inventory, caregiver preparedness

## Abstract

**Background:**

Family hardiness is a key variable contributing to positive family functioning, which has significant effects on the quality of life and the mental health of patientsand caregivers. The factors that contribute to family hardiness support both the psychological and physical well-being of caregivers is unknown. More specifically, the relationship of family hardiness with attachment and caregiver preparedness has not been explored.

**Aim:**

The current study aimed to investigate the family hardiness in caregivers of breast cancer patients and explore the relationship with attachment and caregiver preparedness and identify the associated factors.

**Methods:**

This cross-sectional correlational study was conducted from March to July, 2022. 140 caregivers of breast cancer patients were recruited in two IIIA-grade hospitals in Hunan Province using convenience sampling. Data were collected using a personal characteristics questionnaire, The Family Hardiness Index (FHI), Caregiver Preparedness Scale (CPS), and the Experiences in Close Relationships Inventory-Revised Edition (ECR-R). Chi-square, Pearson’s correlation coefficient, generalized additive model and multiple logistic regression analyses were performed.

**Results:**

A total of 140 caregivers participated in the study. The mean age of participants was (42.29 ± 14.54) years and most of them were male (57.1%). The mean FHI score of caregivers was 58.11 ± 5.67. Multiple linear regression analysis indicated that family hardiness is affected by ECR-R, CPS, education level, and knowledge of breast cancer. The score of CPS was positively associated with family hardiness (*β* = 0.265, *p* < 0.001), whereas ECR-R negatively predicted family hardiness (*β* = −0.078, *p* < 0.001).

**Conclusion:**

Family hardiness plays a critical role in helping caregivers manage the stresses associated with providing care to breast cancer patients. Enhancing caregiver preparedness and education, as well as addressing attachment-related issues, can significantly improve family hardiness. In light of our findings, we suggest that closer relationships within families, adding preparedness and knowledge of disease should be encouraged during the care of breast cancer patients.

## 1 Introduction

Breast cancer constitutes a progressively prevalent malignancy worldwide, posing a significant threat to women health (1). As elucidated in the 2018 China Cancer Center report, the global incidence of breast cancer cases and associated fatalities stands at 11.2 and 9.2%, respectively (2). With the rapid evolution of diagnostic and therapeutic methodologies, the 5-year relative survival rate for breast cancer patients now approximates 73% (3). Nevertheless, individuals post-surgery encounter an array of challenges, necessitating protracted rehabilitation processes, during which manifestations such as alterations in body image, sexual dysfunction, shifts in social roles, the specter of cancer recurrence and metastasis, as well as the burden of anxiety and fear, may ensue (4).

Family caregivers, often referred to as informal caregivers, encompass spouses, partners, children, friends, relatives, and neighbors (5). The intricacies of the illness afflicting the cared-for individual frequently render family caregivers insufficiently prepared. Previous research has indicated that suboptimal adjustment to caregiving roles, deficient psychological readiness and self-assurance, coupled with adverse emotions such as anxiety, depression, and tension, can culminate in physical and mental health detriments for family caregivers (6). Moreover, these negative emotional states may contribute to inadequate caregiving measures, such as poor medication management, lack of emotional support, and neglect of daily care routines, hastening the deterioration of the patient’s condition and compromising the quality of life for both the patient and the caregiver (7). Family caregivers play a pivotal role in overseeing the recovery process of individuals affected by breast cancer, thus bearing significant responsibilities (8). Nevertheless, studies have demonstrated that caregivers within the familial context of cancer patients undergo an array of psychological, physical, and social stressors throughout the caregiving journey (9).

According to the theory of family hardiness put forward by McCubbin HI (10), if the family can fully exercise its characteristics in the face of pressure or crisis, and make good use of its protective qualities, then it can successfully and smoothly navigate through the crisis and reach a better level of adaptation. Family hardiness refers to the ability of the family to maintain a balanced and stable state throughout its overall efforts in the face of pressure or crisis (11). Researchers have summarized various internal and external factors that promote hardiness of the families of cancer patients, but most of them are qualitatively oriented, so it is difficult to identify which factors can lead to specific and effective protective outcomes (12).

Elevated experiences in close relationships inventory-revised edition (ECR-R) scores denote heightened levels of attachment anxiety or avoidance. Fagundes CP et al. (13) demonstrated that elevated levels of anxious and avoidant attachment serve as risk factors for the low quality of life among cancer patients. Tao L et al. (14) similarly established that discerning attachment patterns aids caregivers in comprehending patient responses to cancer, treatment adherence, and adaptation. This, in turn, facilitates the development of targeted and individualized cancer psychotherapy interventions or nursing practices, thereby enhancing both the physical and mental health outcomes of patients. While the ECR-R has been applied in the context of cancer patients, to the best of our knowledge, its relationship with family hardiness has yet to be explored in caregivers of individuals affected by breast cancer.

Caregiver preparedness, defined as the state of readiness to provide necessary care to the patient, is a crucial determinant of how effectively caregivers can handle their responsibilities. High levels of preparedness are associated with better mental and physical health outcomes for both caregivers and patients. Research has shown that caregivers who feel more prepared experience less strain and are more resilient in their caregiving roles. Previous research has shown that caregiver preparedness is one of the most important indicators for evaluating quality of care (15).

Family hardiness is crucial not only for breast cancer patients but also for their caregivers, who play a pivotal role in the recovery process. Identifying the factors affecting family hardiness is urgent because it can enhance the well-being and resilience of both patients and caregivers. Despite the recognized importance of family hardiness, caregiver preparedness, and attachment styles in caregiving, there is a notable gap in the literature regarding their interrelationships, especially among caregivers of breast cancer patients. Current studies have predominantly focused on qualitative aspects, making it difficult to identify specific and effective protective outcomes for family hardiness. To address this gap, our study aims to: Evaluate Family Hardiness: Assess the level of family hardiness among primary caregivers of breast cancer patients using the Family Hardiness Index (FHI). Examine Caregiver Preparedness: Measure the preparedness of these caregivers using the Caregiver Preparedness Scale (CPS) and determine its relationship with family hardiness. Assess Attachment Styles: Utilize the Experiences in Close Relationships Inventory-Revised (ECR-R) to identify the attachment styles of caregivers and analyze their impact on family hardiness. Identify Influencing Factors: Conduct multiple linear regression analysis to identify the factors influencing family hardiness among caregivers, considering sociodemographic variables, caregiver preparedness, and attachment styles.

By systematically exploring these objectives, this study seeks to provide a comprehensive understanding of the factors affecting family hardiness in caregivers of breast cancer patients. The findings aim to inform the development of targeted interventions to support caregivers, enhance family hardiness, and improve the quality of life for both patients and their caregivers.

## 2 Methods

### 2.1 Study design and subjects

From March to July 2022, breast cancer patients receiving surgery in two IIIA-grade hospitals in Hunan Province were selected by convenience sampling. These hospitals, while not exclusively specialized in cancer treatment, have dedicated oncology departments and substantial experience in handling breast cancer cases. The selection of these hospitals was based on convenience sampling to ensure the feasibility and manageability of data collection within the specified timeframe. Primary family caregivers, aged at least 18, willing to participate, were included. Primary caregivers, defined as individuals who are the main providers of physical and emotional support to patients, play a crucial role in the recovery process of breast cancer patients following surgery. These caregivers, often family members, are pivotal in ensuring the well-being of the patient during the rehabilitation phase. No caregivers were recruited from the community. There were no specific restrictions based on the prognostic stages of cancer, allowing the inclusion of caregivers of patients at various stages of breast cancer recovery. For respondents who were illiterate or unable to read, a researcher verbally presented the questions and recorded the responses. Individuals incapable of communication or questionnaire response due to physical or mental illness (e.g., delirium or dementia) were excluded. Data were collected from caregivers. All participants provided signed informed consent, submitted to the ethics committee for approval.

Sample size was calculated according to the cross-sectional study sample size calculation formula (16) *n* = (U*α*S/*δ*) ^2^. According to the preliminary test, the standard deviation *S* = 5.6, taking the allowable error *δ* = 1, *α* = 0.05, then U*α* = 1.96, and obtaining *n* = 120; considering the dropout rate of 10–15%, the sample size is at least 132.

### 2.2 Data collection

Data were collected using the self-designed general information questionnaire. Various sociodemographic data were recorded, including gender, marital status, age, education level, work status, place of residence, income, relationship with the patient, self-health status, history of caregiving, others need to care, knowledge of breast cancer, duration of care, caring time per day and insurance. Data collection was conducted over a four-month period, from March to July 2022 during the daytime hours. Patients who had undergone breast cancer were identified through the hospital and Family caregivers were primarily responsible for providing care to the patient.

#### 2.2.1 FHI

The Family Hardiness Index (17) (FHI) consists of 20 items assessing caregiver perception, regarding: (1) how collaborative the family members are in the face of hardship, with questions such as, “we get stronger when we encounter big problems” (eight items, commitment); (2) the family’s tendency to deal with stressful situations, with questions including, “my family is boring because we repeat the same activities” (to be coded reversely, six items, challenge); and (3) the sense of control the family perceives collectively, with questions such as, “most of the inconveniences are due to misfortune” (six items, control). Respondents provided their ratings using a four-point scale, from 0 (“not at all”) to 3 (“very much so”). Cronbach’s alpha was 0.8232 for the original scale (18), and 0.85 for the current study.

#### 2.2.2 ECR-R

The Experiences in Close Relationships Inventory-Revised Edition (ECR-R), in its Chinese version, served as the instrument for gaging participants’ attachment relationships (19). This inventory encompasses two dimensions: attachment anxiety, delineating a fear of rejection and abandonment, and attachment avoidance, depicting a reluctance to approach and rely on others. The ECR-R contained 36 entries in total (19). These included attachment anxiety (fear of being rejected; free and abandoned) and attachment avoidance (dislike dependence on others; do not like to be close to others). Each item was graded from 1 (strongly disagree) to 7 (strongly disagree). In this study, the overall Cronbach’s alpha was 0.78, and for the two dimensions, the values of the same coefficient were 0.88 and 0.81, respectively.

#### 2.2.3 CPS

Caregiver preparedness scale (CPS), proposed by American scholar Archbold PG (20), refers to nursing staff ‘s perceived preparedness for the multifaceted tasks and requirements of their care role, which include providing physical care and emotional support, as well as coping with care-related stress. CPS is an instrument for evaluating the preparedness of caregivers who assist patients with chronic conditions (20). It contains eight items and uses a five-point Likert scale for responses ranging from 0 (“not at all prepared”) to 4 (“very well prepared”). Thus, the components of the CPS investigate the extent to which a caregiver feels prepared to take care of both the physical and emotional needs of a patient, with a higher score meaning higher preparedness. The validity and reliability of the CPS have been demonstrated for caregivers of patients with heart failure, showing suitable goodness-of-fit indices in confirmatory factor analysis (e.g., comparative fit index, 0.97; root mean square error of approximation, 0.065) and supportive reliability (Cronbach’s alpha = 0.91) (21).

The original versions of the ECR-R, FHI, and CPS were translated into Chinese through a rigorous forward-backward translation procedure (22, 23). This involved independent translations by bilingual experts, synthesis into a single version, and back-translation by a separate group of bilingual experts. Discrepancies between the back-translated and original versions were resolved to ensure semantic equivalence. To validate the content, a panel of oncology nursing and psychology experts reviewed the translated questionnaires. Pilot testing on a small sample of caregivers was conducted to ensure clarity and cultural appropriateness. Finally, the translated questionnaires were administered to a larger sample to assess reliability (Cronbach’s alpha) and construct validity (factor analysis).

### 2.3 Statistical analyses

The data were subjected to analysis utilizing SPSS 22.0 and Stata 17.0 software. Descriptive statistics data are articulated in terms of frequency and percentage. Family hardiness, experiences in close relationships inventory, and caregiver preparedness scores are presented as mean values accompanied by standard deviations. *T*-tests or ANOVA analyses were employed to compare family hardiness scores among breast cancer patients with different characteristics. Pearson correlation analysis was utilized to assess associations between family hardiness and attachment, as well as caregiver preparedness. Factors with a *p*-value less than 0.05 in the univariate regression analysis were included in the multivariate regression. Self-health status was measured using a self-reported item in the questionnaire where caregivers rated their overall health on a 5-point Likert scale ranging from 1 (very poor) to 5 (very good). History of caregiving means caregiving to breast cancer patients. Others need to care means he needs to care not only for breast cancer patients, but also for other families, such as other sick people. Knowledge of breast cancer was measured using a structured questionnaire that included items on breast cancer symptoms, treatment options, and care procedures. Caregivers were asked to respond to multiple-choice and true/false questions to assess their understanding of the disease. Duration of care means how long it has been taking care of. Lastly, multiple linear regression analysis was applied to explore the factors influencing family hardiness. Both continuous and categorical variables were included. Categorical variables were incorporated into the multiple linear regression model using dummy coding. All tests were two-tailed, and significance was set at *p* < 0.05.

## 3 Results

### 3.1 General characteristics of the study subjects

In this study, 140 subjects were finally included. As shown in Table 1, of the total number of respondents, 80 (57.1%) of the study subjects were male. The age lower than 40 years old was 65 (46.4%), 25 (17.9%) cargivers were single. 44 (31.4%) were educated at university or above, and monthly income in caregivers were most in 3,000 to 4,999 RMB (52, 37.1%). In addition, the relationship with patient were mostly spouse (64, 45.7%), most of the caregivers had no history of caregiving (81, 57.9%) and a little knowledge of breast cancer (94, 67.1%). FHI was statistically different between different marital status, age, education level, monthly income, relationship with patient, self-health status, history of caregiving and knowledge of breast cancer with *p*-values less than 0.05.

**Table 1:** Examining family hardiness scores across varied sociodemographic characteristics in caregivers of breast cancer patient.

Variable	*N* (%)	Family hardiness scores (mean ± SD)	*t*/*F*	*p*
Sex			−1.900	0.060
Male	80 (57.1)	57.32 ± 5.44		
Female	60 (42.9)	59.15 ± 5.88		
Marital status			2.305	0.023*
With spouse	115 (82.1)	57.60 ± 5.52		
Single	25 (17.9)	60.44 ± 5.85		
Age (years)			5.425	0.001*
<40	65 (46.4)	60.18 ± 5.78		
≥40, <60	63 (45.0)	56.52 ± 4.78		
≥60	12 (8.6)	55.17 ± 5.80		
Education level			5.062	0.002*
Primary school and below	7 (5.0)	53.43 ± 1.90		
Middle school	44 (31.4)	56.57 ± 5.40		
High school or junior college	45 (32.1)	58.31 ± 5.45		
University or above	44 (31.4)	60.18 ± 5.78		
Work status			0.594	0.710
Not working/retirement	17 (12.1)	58.59 ± 4.68		
Working	123 (87.9)	58.04 ± 5.81		
Place of residence			1.961	0.052
Town	76 (54.3)	58.96 ± 5.59		
Village	64 (45.7)	57.09 ± 5.63		
Monthly income (RMB)			4.013	0.004*
<1,000	14 (10.0)	56.14 ± 4.70		
1,000–2,999	24 (17.1)	56.17 ± 5.61		
3,000–4,999	52 (37.1)	57.37 ± 5.51		
5,000–10,000	32 (22.9)	59.63 ± 4.51		
>10,000	18 (12.9)	61.67 ± 6.88		
Relationship with patient			5.599	0.005*
Child	57 (40.7)	59.32 ± 5.41		
Spouse	64 (45.7)	56.44 ± 5.10		
Friend or relatives	19 (13.6)	60.11 ± 6.88		
Self-health status			3.805	0.025*
Worse	6 (4.3)	57.00 ± 7.77		
Fair	71 (50.7)	56.93 ± 5.07		
Good	63 (45.0)	59.54 ± 5.86		
History of caregiving			0.715	0.017*
Yes	59 (42.1)	59.44 ± 5.79		
No	81 (57.9)	57.14 ± 5.41		
Others need to care			1.066	0.288
Yes	56 (40.0)	57.48 ± 6.16		
No	84 (60.0)	58.52 ± 5.32		
Knowledge of breast cancer			6.539	0.002*
Incomprehension	20 (14.3)	54.35 ± 4.88		
A little	94 (67.1)	58.36 ± 5.31		
Very familiar	26 (18.6)	60.08 ± 6.34		
Duration of care (months)			0.051	0.985
≤6	101 (72.1)	58.18 ± 5.35		
7–12	23 (16.4)	57.92 ± 6.13		
13–24	7 (5.0)	57.43 ± 8.18		
≥24	9 (6.4)	58.33 ± 6.82		
Caring time per day (hours)			0.150	0.861
4–8	61 (43.6)	58.21 ± 5.12		
8–12	39 (27.9)	57.69 ± 6.16		
≥12	40 (28.6)	58.35 ± 6.09		
Insurance			1.977	0.053
Not have	31 (22.1)	56.29 ± 6.00		
Have	109 (77.9)	58.62 ± 5.49		

RMB, Renminbi; SD, Standard Deviation; * (e.g., *p* < 0.05).

### 3.2 The score of family hardiness, ECR-R, and caregiver preparedness scale in study subjects

The results for Family hardiness, ECR-R, and Caregiver Preparedness Scale scores are presented in Table 2. For the study subjects, the mean family hardiness score was 58.11 (SD = 5.67) for the three dimensions examined: commitment (27.06 ± 3.46), control (16.71 ± 2.54), and challenges (14.34 ± 1.48). On average, caregivers demonstrate a moderate level of family hardiness indicated that most caregivers possess a fair degree of commitment to their roles, a sense of control over caregiving situations, and an ability to view caregiving challenges as opportunities for growth. However, it also implies that there is room for improvement, as some caregivers may struggle with fully harnessing these internal resources to manage stress and maintain family stability. ECR-R, including attachment avoidance and attachment anxiety, the mean score was 55.49 ± 14.50 and 66.18 ± 16.40, respectively, sugggestedmoderate levels of these attachment characteristics among caregivers. Moderate attachment anxiety indicates that caregivers may have concerns about rejection or abandonment, which could affect their emotional stability and caregiving behavior. Similarly, moderate attachment avoidance suggests that some caregivers may be uncomfortable with closeness and dependency, potentially impacting their ability to provide emotional support to the patient. Additionally, the mean caregiver preparedness score was 22.31 (SD = 5.69), indicated that caregivers generally feel adequately prepared to handle caregiving duties but may experience some challenges. While caregivers possess essential skills and knowledge, they might benefit from additional support and resources to enhance their caregiving effectiveness.

**Table 2:** The score of family hardiness, experiences in close relationships inventory, and caregiver preparedness scale in caregivers of breast cancer patient.

Variable	Standard score	Actual score	Mean score (mean ± SD)
Family hardiness	20–80	44–76	58.11 ± 5.67
Commitment	9–36	14–36	27.06 ± 3.46
Control	6–24	10–23	16.71 ± 2.54
Challenge	5–20	11–19	14.34 ± 1.48
Experiences in close relationships inventory	36–252	49–184	121.7 ± 25.01
Attachment avoidance	18–126	19–100	55.49 ± 14.50
Attachment anxiety	18–126	29–110	66.18 ± 16.40
Caregiver preparedness scale	0–32	0–32	22.31 ± 5.69

### 3.3 Correlation between family hardiness and experiences in close relationships inventory and caregiver preparedness scale in caregivers of breast Cancer patients

As can be seen in Table 3, the Pearson’s correlation analysis indicated that family hardiness was negatively correlated with attachment anxiety (*r* = −0.461), indicating that higher levels of attachment anxiety are associated with lower family hardiness. A negative correlation was also found with attachment avoidance (*r* = −0.318). This indicates that higher levels of attachment avoidance are associated with lower family hardiness, suggesting that caregivers who experience higher anxiety about their relationships may struggle more with family resilience. However, a positive correlation was observed with caregiver preparedness (*r* = 0.369), indicating that higher levels of preparedness are associated with greater family hardiness. In all three cases, *p* < 0.01.

**Table 3:** Correlation between family hardiness and experiences in close relationships inventory and caregiver preparedness scale in caregivers of breast cancer patient.

Variable	Family hardiness			
	Commitment	Control	Challenge	Total
Experiences in close relationships inventory				
Attachment avoidance	−0.400**	−0.247**	−0.406**	−0.461**
Attachment anxiety	−0.092	−0.432**	−0.261**	−0.318**
Total	−0.292**	−0.428**	−0.407**	−0.476**
Caregiver preparedness scale	0.368**	0.106	0.369**	0.369**

** (e.g., *p* < 0.01).

### 3.4 Multiple linear regression analysis of factors associated with family hardiness in caregivers of breast Cancer patients

Overall, marital status, age, education level, monthly income, relationship with patient, self-health status, history of caregiving, knowledge of breast cancer, experiences in close relationships inventory, and caregiver preparedness were included in the multiple linear regression model (per formed stepwise), while family hardiness was a dependent variable. The results of this analysis are displayed in Table 4, which showed that total score of caregiver preparedness scale, education level, and knowledge of breast cancer could positively associated with family hardiness (*β* = 0.266, 0.196, and 0.146, respectively). Caregivers who are well-prepared, educated, and knowledgeable about breast cancer are better equipped to handle the challenges of caregiving, contributing to stronger family hardiness. In addition, total score of the experiences in close relationships inventory was negatively predict family hardiness, *β* = −0.345. The total explanatory quantity of the four variables was 33.4%, *F* = 14.968. It shows the proportion of variance in the dependent variable that is explained by the independent variables.

**Table 4:** Multiple linear regression analysis of factors associated with family hardiness in caregivers of breast cancer patient. ^a^

Variable	*B*	*SE*	*β*	*t*	*p*
Total score of the experiences in close relationships inventory	−0.078	0.017	−0.345	−4.535	0.000
Total score of caregiver preparedness scale	0.265	0.072	0.266	3.689	0.000
Education level	1.221	0.484	0.196	2.524	0.013
Knowledge of breast cancer	1.445	0.714	0.146	2.023	0.045

a *R2 = 0.334, F = 14.968.*

**Figure 1:**
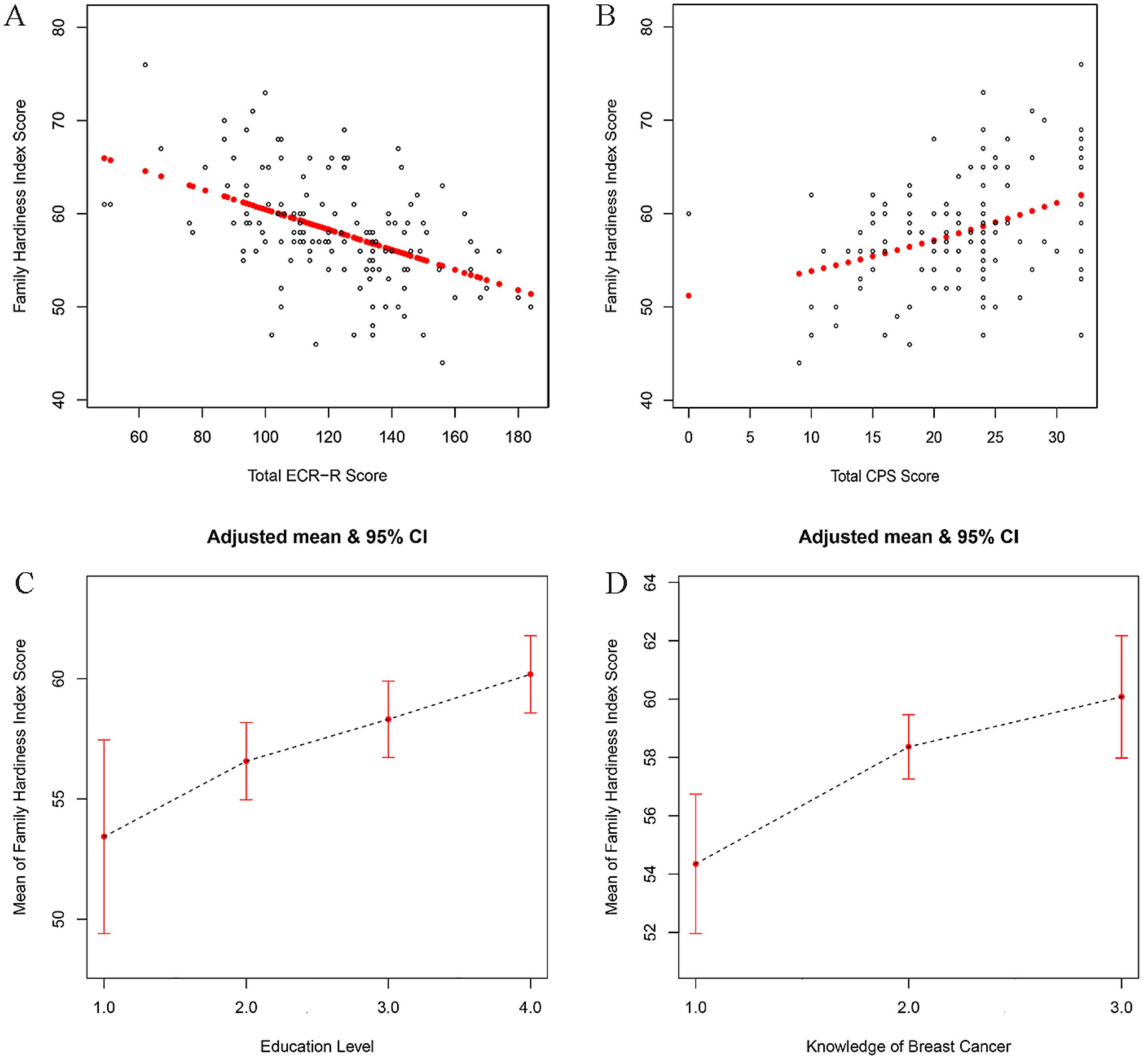
Association of total score of the experiences in close relationships inventory (A), total score of caregiver preparedness scale (B), education level (C), knowledge of breast cancer (D) with family hardiness using smooth spline curves after adjusting multivariate rates. ECR-R, the Experiences in Close Relationships Inventory-Revised Edition; CPS, Caregiver Preparedness Scale; Education level 1 = Primary school and below, 2 = Middle school, 3 = High school or junior college, 4 = University or above; Knowledge of breast cancer 1 = Incomprehension, 2 = A little, 3 = Very familiar.

To visualize the relationships, restricted cubic splines were used for flexible modeling (Figure 1). A linear relationship existed between total score of the experiences in close relationships inventory (Figure 1A), total score of caregiver preparedness scale (Figure 1B), education level (Figure 1C), knowledge of breast cancer (Figure 1D) and family hardiness.

## 4 Discussion

In this study, we explored the current landscape of family hardiness for caregivers in breast cancer patients and investigated its influencing factors. The results revealed moderate family hardiness, with the main contributing factors being caregiver preparedness, education level, knowledge of breast cancer and the experiences in close relationships inventory Family hardiness was positively predicted by total score of caregiver preparedness scale, education level, and knowledge of breast cancer, but negatively predicted by total score of the experiences in close relationships inventory.

In this investigation, caregivers of breast cancer patients demonstrated a family hardiness score of 58.11 (SD = 5.67), aligning with the findings of Cui P (24). In accordance with crisis theory, adverse life events have the potential to impede the fulfillment of fundamental needs among family members, thereby influencing family hardiness. If a family fails to confront the challenges posed by negative events, it may manifest as an imbalance or disharmony, ultimately culminating in family crisis or collapse. The presence of cancer in a family member has multifaceted repercussions, precipitating varying degrees of family crisis and significantly impacting overall familial harmony (25). This elucidates the observed lower family hardiness score among cancer patients in this study, underscoring the importance for nursing staff to be attuned to family hardiness when engaging with breast cancer patients and their families. Such attention can facilitate the restoration of each family to equilibrium and harmony in a timely manner.

The educational attainment of the primary caregiver serves as an indicator of their level of education, reflecting, to some extent, the cultural milieu of the entire family and thereby indicating the family’s capacity to navigate through crisis events. A higher level of education implies caregivers who are more seasoned and adaptable in comparison to those with lower education. Such caregivers possess independent access to disease-related knowledge and exhibit more effective communication with the patient (26). Highly educated caregivers, from their perspective, are inclined to assume responsibility and proactively respond to negative events, thereby preserving a stable family state, exerting control over the unfolding situation, and consequently enhancing family hardiness. From the standpoint of other family members, highly educated caregivers are better equipped to uphold the stability of the family environment, promptly address family conflicts and contradictions, and attend to the particulars of the patients’ lives. Consequently, when confronted with crisis events, highly educated caregivers are better positioned to leverage their internal family resources, adapting to and coping with the stressful circumstances more effectively (27).

The degree to which caregivers are informed about the patient’s illness plays a pivotal role in determining the level of stress experienced by family members, subsequently influencing family hardiness (28). Given the specific nature of breast cancer, patients often bear a substantial psychological burden and emotional tumult, frequently accompanied by negative psychological experiences (29). Families possessing a thorough understanding of the disease exhibit sufficient psychological preparedness and high coordination with treatment, resulting in comparatively lower psychological pressure on both the patient and the primary caregiver. Conversely, families lacking comprehension of the patient’s disease state may tend to overestimate or underestimate the severity, consequently impacting treatment efficacy (30). Simultaneously, primary caregivers are required to actively engage in adjuvant treatments, thereby heightening the demands on their hardiness and cognitive capacity related to the disease. This, in turn, exerts an influence on the psychological well-being and domestic environment of family members. Consequently, a lower level of caregivers’ knowledge about the patient’s disease corresponds to a diminished level of family hardiness.

Furthermore, caregiver preparedness pertains to the state of readiness to deliver requisite care to the patient and serves as the evaluative metric for predicting the strain associated with the caregiving role (31). Serving as the primary support and aide for the patient’s physical and mental needs, the caregiver’s own health may undergo negative repercussions after an extended period of caregiving. Moreover, the caregiver’s physical and mental well-being directly influences the efficacy of the patient’s rehabilitation. In this study, we employed the Caregiver Preparedness Scale, another methodology validated for reliability and validity, to gage the preparedness of individuals providing support to breast cancer patients. This scale has undergone validation for use among caregivers and is a common tool in research involving this demographic.

We also employed the Experiences in Close Relationships Inventory, another validated approach, to assess the status of family intimacy (32). This is currently the most commonly used scale when assessing adult attachment, and it is also the most widely employed scale in research relating to caregiver attachment. When it is applied to family caregivers of cancer patients, some researchers consider that since cancer patients face life-threatening situations—and their caregivers genuinely worry about losing each other—this scale does not accurately reflect the caregiver’s attachment. However, the Cronbach’s alpha coefficients for the attachment anxiety and attachment avoidance subscales in the revised questionnaire were 0.88 and 0.86, respectively. This study found that the experiences in close relationships inventory negatively predicted family hardiness. Individuals with attachment avoidance and anxiety are extremely sensitive in the face of setbacks or threats (33). They tend to overactivate their own emotional regulation strategies and their desire for excessive attention and support, but patients may ignore the psychological state of their caregivers due to their illness. These findings underscore the importance for healthcare professionals to enhance their assessment of the attachment types among caregivers of cancer patients and guide them in adopting positive self-emotional regulation strategies.

This study offers unique insights into the factors influencing family hardiness among caregivers of breast cancer patients. Specifically, it highlights the critical roles of caregiver preparedness, educational level, and knowledge about breast cancer in enhancing family resilience. These findings contribute to the existing knowledge by emphasizing the importance of psychological and educational support for caregivers, which has not been extensively explored in previous research. Furthermore, the study underscores the predictive value of attachment styles in understanding family hardiness, providing a novel perspective on the emotional dynamics within caregiving families.

### 4.1 Limitations

There are several limitations of this study that must be acknowledged. First, the sample size is relatively limited, and sample collection should be expanded in future research to test the reliability of the present results. Second, the cross-sectional design of this study means that its conclusions regarding causality remain controversial, necessitating longitudinal studies to verify these findings. Third, several factors that could influence the results were not measured, including the psychological resilience of caregivers, the social support network, the severity of patients’ symptoms, and the specific cultural context of caregiving. Additionally, the use of convenience sampling limits the generalizability of the findings, as the sample may not be representative of the broader population of caregivers. Regrettably, the prognostic stages of breast cancer, type of surgery (e.g., lumpectomy or mastectomy), functional status of patients data are no longer available.

## 5 Conclusion

In conclusion, this study underscores the significant role of caregiver preparedness, education, and disease knowledge in enhancing family hardiness among caregivers of breast cancer patients. These findings have important policy and research implications. Policymakers should consider developing structured training programs for caregivers, focusing on mental and physical health management as well as comprehensive disease knowledge. From a research perspective, future studies should explore the longitudinal impact of these factors on family resilience and investigate additional variables such as social support and cultural context. Implementing these recommendations could lead to better support systems for caregivers and improved outcomes for both patients and their families.

## Data Availability

The raw data supporting the conclusions of this article will be made available by the authors, without undue reservation.
